# Assessment of normalized cerebral blood flow and its connectivity with migraines without aura during interictal periods by arterial spin labeling

**DOI:** 10.1186/s10194-021-01282-y

**Published:** 2021-07-14

**Authors:** Di Zhang, Xiaobin Huang, Cunnan Mao, Yuchen Chen, Zhengfei Miao, Chunmei Liu, Chenjie Xu, Xinying Wu, Xindao Yin

**Affiliations:** 1 Department of Radiology, Nanjing First Hospital, Nanjing Medical University, No.68, Changle Road, Nanjing, 210006 Jiangsu China; 2 Department of Neurology, Nanjing First Hospital, Nanjing Medical University, No.68, Changle Road, Nanjing, 210006 Jiangsu China; 3 Department of Pain Treatment, Nanjing First Hospital, Nanjing Medical University, No.68, Changle Road, Nanjing, 210006 Jiangsu China

**Keywords:** Migraine without aura, Arterial spin labeling MRI, Perfusion imaging, Regional cerebral blood flow

## Abstract

**Background:**

Migraine constitutes a global health burden, and its pathophysiology is not well-understood; research evaluating cerebral perfusion and altered blood flow between brain areas using non-invasive imaging techniques, such as arterial spin labeling, have been scarce. This study aimed to assess cerebral blood flow (CBF) and its connectivity of migraine.

**Methods:**

This study enrolled 40 patients with episodic migraine without aura (MwoA), as well as 42 healthy patients as control (HC). Two groups of normalized CBF and CBF connectivity were compared, and the relationship between CBF variation and clinical scale assessment was further evaluated.

**Results:**

In comparison to HC subjects, MwoA patients exhibited higher CBF in the right middle frontal orbital gyrus (ORBmid.R) and the right middle frontal gyrus, while that in Vermis_6 declined. The increased CBF of ORBmid.R was positively correlated with both the Visual Light Sensitivity Questionnaire-8 (VLSQ-8) and the monthly attack frequency score. In MwoA, significantly decreased CBF connectivity was detected between ORBmid.R and the left superior frontal gyrus, the right putamen, the right caudate, as well as the right angular gyrus. In addition, increased CBF connectivity was observed between the left calcarine cortex and ORBmid.R.

**Conclusions:**

Our results indicate that migraine patients exhibit abnormalities in regional CBF and feature CBF connection defects at the resting state. The affected areas involve information perception, information integration, and emotional, pain and visual processing. Our findings might provide important clues for the pathophysiology of migraine.

## Background

For the period 1990–2017, migraine, a prevalent primary headache disorder, was considered the second most frequent factor associated with disability-adjusted life years according to the Global Burden of Disease study [1]. Epidemiological studies estimate that migraine affects 15 to 25% of women and 6 to 8% of men worldwide [2]. The condition is characterized by recurrent throbbing headache attacks, often accompanied by nausea, vomiting, photophobia, phonophobia, or allodynia [1, 2]. Despite its significance, the pathogenesis of migraine has not been fully established. Advanced structural and functional imaging studies have provided some clues for understanding the pathophysiology of migraine and migraine-related dysfunction. The trigeminal neurovascular hypothesis is a comprehensive hypothesis that has played a leading role in explaining the pathogenesis of migraine [3]. Triptan drugs are clinically effective anti-migraine drugs developed according to this hypothesis. Triptans only have a slight constriction effect on blood vessels under physiological conditions, but can make abnormally dilated blood vessels and meningeal blood vessels constrict significantly [4]. The visual cortex, the limbic system, as well as pain and cognitive networks are also involved in the process of migraine [5–10].

According to the trigeminal neurovascular theory, changes in cerebral blood flow might occur during migraine attacks. Thus far, some studies have reported on cerebral perfusion changes in migraine patients. These are mostly case reports and tend to concentrate on migraine patients with aura. Research on perfusion imaging include the widely applied techniques of positron emission tomography (PET), CT perfusion, single photon-emission computed tomography (SPECT), dynamic susceptibility contrast (DSC), magnetic resonance perfusion imaging and perfusion-weighted imaging (PWI) [11–13]. One of the non-invasive cerebral perfusion imaging techniques is arterial spin labeling (ASL), that can be utilized to quantitatively assess the degree of cerebral perfusion without having to apply a gadolinium-based contrast agent [14, 15]. The results of ASL are consistent with PET and DSC, and ASL provides a brain CBF measurement with high reliability and repeatability [16, 17]. Pseudo-continuous ASL (PCASL) can be used for whole-brain scanning and has a high clinical application value [14, 15, 18, 19]. Most studies confirmed the detection of cerebral hypoperfusion in the acute phase of aura followed by rebound hyperperfusion [20, 21] and hyperperfusion in the onset phase [22–24]. It has also been found that local resting cerebral blood flow in the lateral hypothalamus decreases prior to migraine onset [25].

To date, few studies have employed ASL imaging to evaluate the interictal perfusion of migraine without aura (MwoA), and their results were not consistent. The presence of hypoperfusion in brain areas was confirmed by ASL MRI during MwoA attacks [26]. A recent study found increased CBF in the ipsilateral dorsolateral pontine during attack in MwoA using PCASL [27]. The ASL MRI method was also used to compare cerebral perfusion during migraine without aura attack and a headache-free period, while no global or regional differences were found [28]. The differences in the above results may be attributed to the fact that they were case reports or included small cohorts, and featured a diversity of disease severity, accompanying disorders, scanning methods or time interval from onset to examination. Considering that the brain is in a continuous internal metabolic activity state at rest, ASL provides the opportunity to detect and monitor changes in tissue perfusion that might indicate brain dysfunction. This study aimed to detect the pattern of CBF at the interictal phase of episodic MwoA and establish the relationships between changes in CBF and clinical scale assessment. In addition, we also investigated whether the abnormal CBF brain regions of MwoA patients also show changes in CBF connections. It is assumed that the links between cerebral perfusion and changes in cerebral blood flow in MwoA patients are different compared to those in healthy controls (HC), and these changes can be associated with a certain assessment scale in the clinical practice.

## Methods

### Subject selection criteria

This study was conducted with the approval of the Ethics Committee of Nanjing First Hospital, Nanjing, China. All subjects signed the informed consent forms. A total of 55 patients were recruited from the pain clinic and the Neurology Department of Nanjing First Hospital between the period of May 2018 to April 2020. All of them had episodic MwoA, according to the International Classification of Headache Disorders (Third Edition, beta version; ICHD-3 beta), and were aged between 18 and 50 years. Based on matching for age, gender and number of years in education 44 HC subjects were finally included in the study.

The following exclusion criteria were applied: (1) Poor image quality; (2) MRI contraindications, (3) neuropsychological disorders; (4) history of alcohol or substance abuse; (5) brain damage or other neurological diseases (such as epilepsy, stroke, and physical disease) that can affect research results, (6) immediate relatives with a history of headache, and (7) take any vasoactive drugs for 1 week before the scan. In addition, to minimize the effect of hormone levels on cortical excitability, MRI scans of all female subjects were performed mid-menstrual cycle with the exception of pregnancy and lactation. Patients were headache free for at least 48 h (before and after the scan), fasted for 4 h, and were not allowed coffee, tea, alcohol, cocoa, and tobacco within 12 h before the start of the study. Such as in our previous study, all patients were assessed by the Self-Rating Anxiety Scale (SAS), Self-Rating Depression Scale, (SDS), the Montreal Cognitive Assessment screening test (MOCA), the Headache Impact Test-6 (HIT-6), the Migraine Disability Assessment Questionnaire (MIDAS), and the Visual Light Sensitivity Questionnaire-8 (VLSQ-8). The latter contains eight questions to assess the presence and severity of visual light sensitivity [28]. All HC subjects were evaluated for SAS, SDS and MOCA scores. No significant differences occurred between the two groups in education level, age, gender, SAS score, SDS score and MOCA score.

### MRI data acquisition

All subjects underwent a PCASL scan on an Ingenia 3.0T MR system (Philips Medical Systems, Netherlands) with the application of a standard eight-channel digital head coil receiver. During the MRI scans, participants wore headphones and earplugs, and lied on their backs. A moderately tight, comfortable foam cushion was used to reduce head movement. In addition, participants were required to rest peacefully and close their eyes. All subjects fasted for at least 4 h. The resting-state perfusion imaging technique was conducted with the application of a PCASL sequence as follows: repetition time = 4000 ms; label duration = 1650 ms; echo time = 11 ms; flip angle = 90°; post-label delay = 1600 ms; field of view = 240 mm × 240 mm; slice thickness = 4 mm with 10% gap; matrix = 64 × 64; 20 axial slices; total scan duration = 4 min 08 s. Finally, each subject contained 60 volumes used as 30 label-control image pairs.

### Analysis of MRI data and calculation of normalized CBF

The ASL image data were analyzed by the Statistical parameter mapping software (SPM8) (https://www.fil.ion.ucl.ac.uk/spm/software/spm8/) and the ASL data processing toolbox ASLtbx (https://cfn.upenn.edu/~zewan). The detailed procedures for the calculation of CBF maps were as described in our previous study [29, 30]. Briefly, according to the motion parameters provided by SPM, participants with a translation greater than 2 mm and a rotation greater than 2° were excluded from the analysis. Frame-wise displacement (FD) was acquired for the between group comparisons. The CBF images were subjected to nonlinear transformation using SPM8, and were co-registered with the PET-perfusion template in Montreal Neurological Institute (MNI) space. Each CBF co-registered in this manner was spatially trimmed to an 8 mm × 8 mm × 8 mm FWHM of the Gaussian curve. The normalization of results was performed by dividing the value of cerebral blood flow per voxel by the average cerebral blood flow through the whole brain. The voxel-size for the normalized CBF map is 2 mm * 2 mm * 2 mm. After data processing, 15 patients and 2 healthy subjects were excluded due to movement and subsequent image distortion, resulting in the final enrolment of 40 patients and 42 HC subjects.

### Comparison of normalized CBF between MwoA and HC

The difference in normalized CBF (default gray matter template) between MwoA and HC was studied using a two-sample t-test. We used 5000 permutations and threshold free cluster enhancement and family wise error rate correction (TFCE (FWER, p < 0.05)) was performed.

### Determination of CBF connectivity

Characterizing CBF concurrent changes across subjects between pairs of brain regions by computing the correlation coefficient is able to provide a CBF connectivity measure among these brain regions. Considering previously used research methods, clusters with significant differences in CBF between groups were selected as regions of interest to detect whether different brain regions featured any abnormal CBF connections that corresponded to changes in CBF [29–31]. The CBF value of each ROI was extracted from the normalized CBF map obtained for each patient. The CBF connection between each ROI and all other voxels in the intact brain was calculated by a multiple regression model for each group, where the confounding covariates were gender, age, education and FD. This step allowed for the identification of voxels that were in either positive or negative correlation with the CBF of each ROI in every group. For each ROI, the CBF connectivity maps of the two groups were merged into a spatial mask, where the normalized CBF of each voxel was correlated with the normalized CBF of the ROI in any of the two groups. To map the voxels that expressed a significantly different CBF correlation with each seed ROI between the MwoA and the healthy subjects, T contrasts were established within the spatial mask of the CBF connectivity map of the ROI after controlling for gender, age, education and FD. TFCE (FDR, p < 0.05) was used to correct multiple comparisons and to evaluate the between-group difference of CBF connectivity.

### Correlation analysis using the evaluation scores

The Kolmogorov–Smirnov test was applied to test the normality of clinical and population baseline data distribution. The two-sample T test (for age) and χ^2^ test (for gender) were used to analyze differences between groups. The Mann–Whitney U test was employed to analyze measurement data with non-normal distribution (i.e., education level, SAS score, SDS score, MoCA score, and VLSQ-8 score). For the correlation analysis, the normalized CBF value of each brain region showing significant differences between the groups was extracted for each subject. The relationships between CBF value and clinical parameters were determined by Spearman correlation analysis, while the age, gender and education level were adjusted. The above statistical analysis was performed using SPSS 19.0 software (version 19.0, SPSS Inc., Chicago, IL, USA). Spm12 software was used to analyze CBF and CBF connectivity between groups. TFCE (FWER, p < 0.05) was used to correct multiple comparisons and to evaluate the between-group difference.

### Statistical analysis

The Kolmogorov–Smirnov test was applied to test the normality of clinical and population baseline data distribution. The two-sample T test (for age) and χ^2^ test (for gender) were used to analyze differences between groups. The Mann–Whitney U test was employed to analyze measurement data with non-normal distribution (i.e., education level, SAS score, SDS score, MoCA score, and VLSQ-8 score). For the correlation analysis, the normalized CBF value of each brain region showing significant differences between the groups was extracted for each subject. The relationships between CBF value and clinical parameters were determined by Spearman correlation analysis, while the age, gender and education level were adjusted. The above statistical analysis was performed using SPSS 19.0 software (version 19.0, SPSS Inc., Chicago, IL, USA). Spm12 software was used to analyze CBF and CBF connectivity between groups. TFCE (FWER, p < 0.05) was used to correct multiple comparisons and to evaluate the between-group difference.

## Results

### Participants and clinical data

Table 1 summarizes the demographic and clinical assessments of the subjects. There is no significant difference in age, gender and education level between the two groups (all *p* > 0.05).

**Table 1:** Demographic and clinical characteristics of participants included in the study

	MwoA patients (n = 40)	Healthy controls (n = 42)	p value
Age (years)	35.10 ± 9.48	41.05 ± 9.95	0.992
Gender (male/female)	10:30	15:27	0.208
MoCA score	26.15 ± 1.39	26.15 ± 1.81	0.817
SAS score	40.00 ± 7.54	40.20 ± 7.78	0.857
SDS score	41.71 ± 9.63	41.49 ± 9.44	0.066
Education (years)	14.18 ± 2.61	13.18 ± 2.91	0.190
FD (mm)	0.15950 ± 0.04398	0.18238 ± 0.07675	0.104
Duration (years)	9.20 ± 5.82	NA	NA
Headache laterality, n (%)		NA	NA
Unilateral	18 (45%)	NA	NA
Bilateral	14 (35%)	NA	NA
Shift	8 (20%)	NA	NA
Frequency (d/m)	5.38 ± 2.36	NA	NA
VAS	5.03 ± 1.53	NA	NA
Mild, n (%)	10 (25%)	NA	NA
Moderate, n (%)	23 (57.5%)	NA	NA
Severe, n (%)	7 (42.5%)	NA	NA
HIT-6 score	56.70 ± 9.54	NA	NA
MIDAS score	10.38 ± 8.03	NA	NA
VLSQ-8 score	21.00 ± 5.48	11.80 ± 2.85	< 0.001

Visual Analogue Scale 0–10: mild 1–3; moderate 4–6; severe 7–10. Measurement data are expressed in mean and standard deviation

*MoCA* Montreal Cognitive Assessment, *SAS* Self-Rating Anxiety Scale, *SDS* Self-Rating Depression Scale, *FD* frame-wise displacement, *HC* healthy control, *HIT-6* Headache Impact Test-6, *MIDAS* the Migraine Disability Assessment Score, *VLSQ-8* Visual Light Sensitivity Questionnaire-8

### Differences between groups in the normalized CBF values for resting state in interictal period

**Fig. 1:**
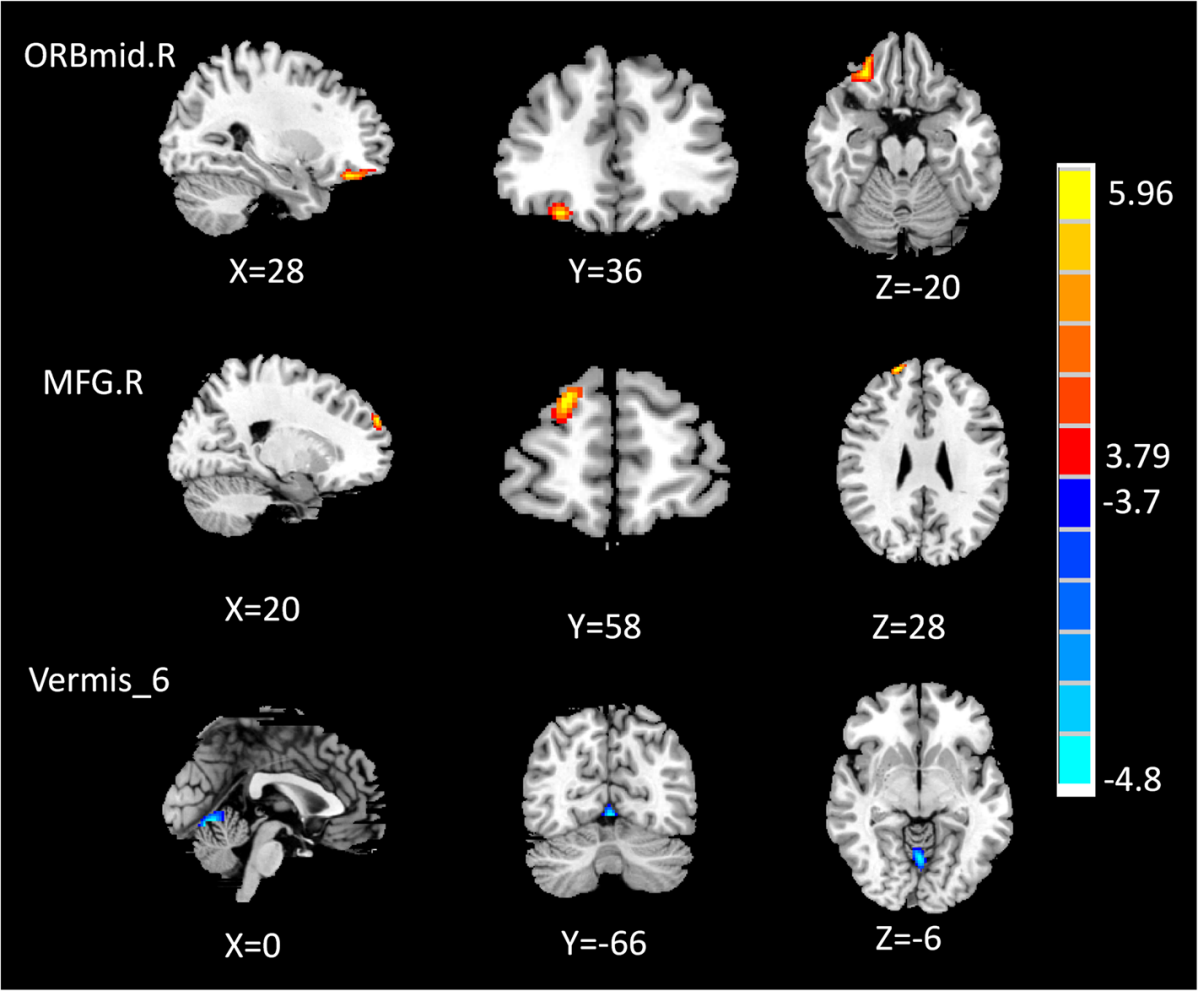
Voxel-based analysis indicates the brain regions with significant group differences in the normalized CBF. Compared with HC, the MwoA patients showed increased CBF in the right middle frontal orbital gyrus (ORBmid.R), right middle frontal gyrus (MFG.R) and decreased CBF in Vermis_6. These findings correspond to TFCE for correct multiple comparisons (FDR corrected, p < 0.05). *MwoA* migraine patients without aura, *HC* healthy control, *ORBmid.R* right middle frontal orbital gyrus, *MFG.R* right middle frontal gyrus

The regional CBF differences between MwoA and HC patients are presented in Fig. 1 and Table 2. The CBF was raised in the right middle frontal orbital gyrus (ORBmid.R), and the right middle frontal gyrus (MFG.R) in MwoA patients compared with HC patients. On the contrary, the CBF in Vermis_6 of these patients was lower than that of the control group.

**Table 2:** Brain regions with significant group differences in normalized CBF

	Brain region	Normalized CBF		Peak MNI coordinates			Voxel size	Peak t score
		MwoA	HC	X	Y	Z		
MwoA > HC	ORBmid.R	1.223 ± 0.163	1.024 ± 0.080	28	36	− 20	155	6.397
	MFG.R	0.919 ± 0.136	0.772 ± 0.105	20	58	28	144	4.547
MwoA < HC	Vermis_6	1.092 ± 0.206	1.332 ± 0.127	0	− 66	− 6	124	− 5.085

Thresholds were set at a corrected p < 0.001 corrected by FDR criterion

*MNI* Montreal Neurological Institute, *MwoA* migraine patients without aura, *HC* healthy control, *ORBmid.R* right middle frontal orbital gyrus, *MFG.R* right middle frontal gyrus

### Group differences in normalized CBF connectivity

**Fig. 2:**
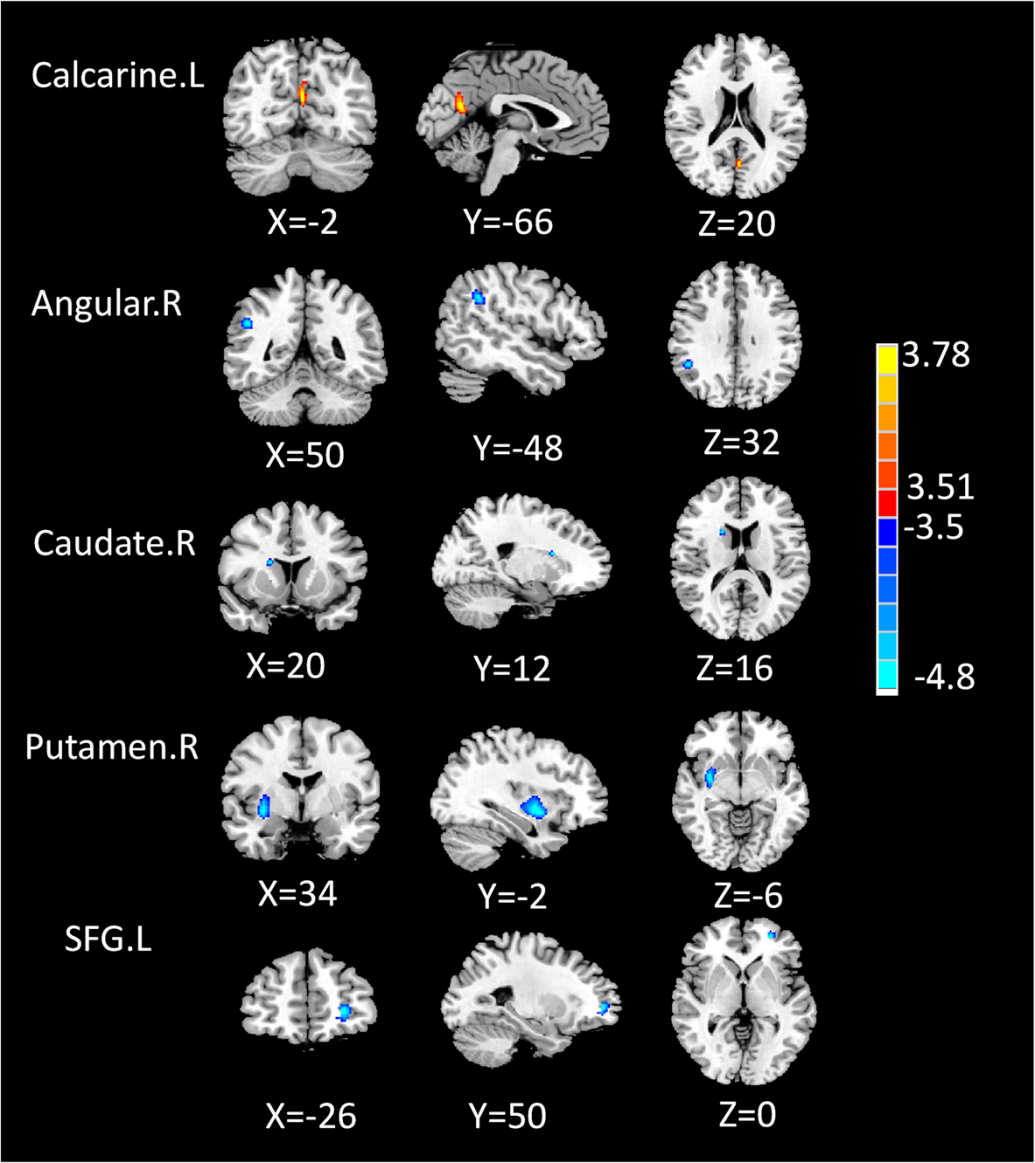
Compared with HC, the MwoA patients exhibited decreased CBF connectivity between the seed ROI of the ORBmid.R and the right putamen, left superior frontal gyrus (SFG.L), right caudate as well as right angular, and increased CBF connectivity between the seed of the ORBmid.R and left calcarine cortex

The differences in CBF connectivity are presented in Fig. 2 and Table 3. In comparison with HC patients, MwoA patients exhibited reduced CBF connectivity between ORBmid.R and the right putamen, the left superior frontal gyrus (SFG.L), the right caudate, as well as the right angular gyrus, and the CBF connectivity between ORBmid.R and the left calcarine cortex was raised. The CBF connection with MFG.R and the Vermis_6 as seed points did not show any significant differences between groups.

**Table 3:** Brain regions with significant group differences in CBF connectivity

ROI	Brain region	Normalized CBF		Peak MNI coordinates			Voxel size	Peak t score
		MwoA	HC	X	Y	Z		
ORBmid.R	Calcarine.L	1.177 ± 0.187	1.171 ± 0.153	− 2	− 66	20	102	3.8408
	Angular.R	0.805 ± 0.113	0.893 ± 0.102	50	− 48	32	72	− 5.1188
	Caudate.R	0.499 ± 0.780	0.478 ± 0.058	20	12	16	17	− 3.9317
	Putamen.R	1.141 ± 0.149	1.074 ± 0.099	34	− 2	− 6	304	− 4.3331
	SFG.L	1.121 ± 0.179	1.049 ± 0.122	− 26	50	0	69	− 4.9305

### Correlations between normalized CBF and clinical scale assessment

**Fig. 3:**
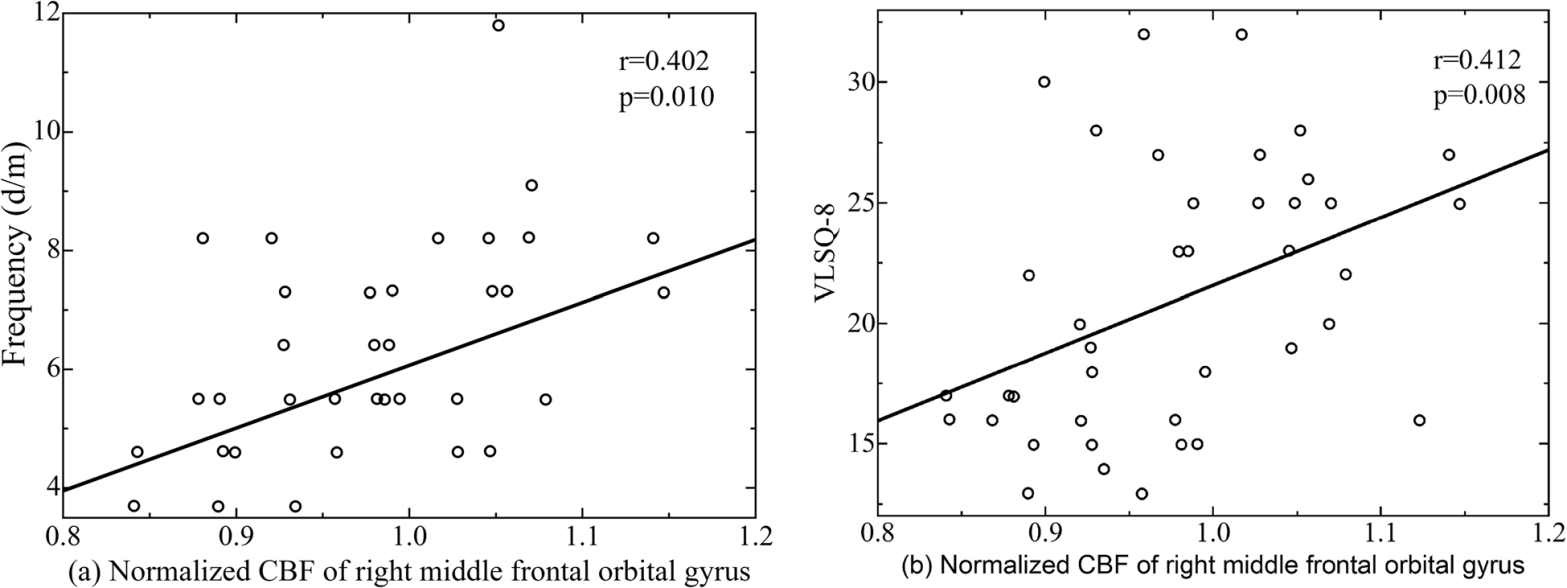
The monthly attack frequency score (**a**) and VLSQ-8 score (**b**) were positively correlated with the normalized CBF of right middle frontal orbital gyrus, respectively (r = 0.402, p = 0.010; r = 0.412, p = 0.008)

The correlation analysis showed that the monthly attack frequency score and the VLSQ-8 score were positively correlated with the increased CBF in ORBmid.R (Fig. 3). Meanwhile, the correlations between further clinical parameters and changes in CBF in the MFG.R and Vermis_6 of MwoA patients (including the SAS score, SDS score, MoCA score, disease course, VAS score, hit6 score, and MIDAS score) proved as statistically insignificant.

## Discussion

This study used 2D PCASL, a type of non-enhanced sequence, to detect changes in cerebral perfusion patterns and CBF connectivity changes of MwoA in the interictal phase. As far as the authors are concerned, this study is the first of its kind to utilize ASL-MRI for establishing the CBF connectivity patterns of MwoA patients. Furthermore, among similar studies using perfusion MRI to obtain perfusion maps of migraine during the interictal phase, our case series features the largest sample size. The trigeminal nerve vascular hypothesis, which is one of the most recognized approaches to describe the pathogenic mechanisms of migraine, states that changes in the blood flow within the brain tissue may be mediated by the secretion of certain vasoactive substances, with varying effects between different brain regions [3, 32].

In our study, increased perfusion was detected in the ORBmid.R and MFG.R, which are key areas of the prefrontal lobe. The orbital frontal cortex (OFC) is considered as a key area for sensory integration, self-control and emotional expression [33, 34]. Patients showed corresponding behavioral changes after OFC injury, such as recognition defects of emotional expression [34]. Chen et al. investigated the regional brain volume changes in episodic migraine, and showed that the right orbital frontal lobe had increased volume [35]. Compared with HC, the ALFF score of the orbital cortex in migraine patients was significantly increased during the interictal period [36]. In non-menstrual phase of primary dysmenorrhea, reduced ReHo values were observed in OFC [37]. We speculated that the increased volume, ALFF value and decreased ReHo value of orbital cortex may indicate the adaptation of the central nervous system, which could enhance descending pain modulation. The basis of these adaptations is the increase in local blood flow. Conversely, these adaptations may lead to increased local blood flow affecting the metabolism of the orbital cortex. This hypothesis can also be explained by the correlation between the attack frequency score and the CBF of ORBmid.R area, as established in our research.

Magnetic resonance imaging technology has been extensively featured in research on the connection changes in patients with migraine, such as the anatomical connection of diffusion tensor imaging, the structural connection of structural MRI, and the functional connection of functional MRI. Nonetheless, to our knowledge, no research has yet investigated the CBF connections of migraine. Although both blood oxygen level dependent (BOLD) connections and CBF connections measure the functional correlation among brain regions, their calculation methods are different and the results also have different physiological meanings. BOLD connectivity can be obtained by measuring the time correlation between BOLD signal fluctuations in various brain regions. Meanwhile, the calculation of CBF correlation coefficient among a group of brain regions yields the CBF connectivity. Although multiple BOLD connectivity values (one value per person) can be obtained from a given dataset, only a single such value can be established of CBF connectivity. CBF connectivity indicates the changes of cerebral blood flow in group level. CBF connections are only regulated by regional CBF, and the physiological mechanism may be more defined than BOLD connections. BOLD connections are affected by hemodynamic parameters, such as cerebral blood volume and cerebral oxygen metabolic rate Animal experiments have shown that OFC selectively connects with other prefrontal lobes (dorsolateral prefrontal cortex and medial prefrontal cortex) and the sensory cortex, including smell, taste, somatosensory, auditory and visual processing centers, and the amygdala [38]. In this study, we detected CBF disconnections between ORBmid.R and the regions of right putamen, SFG.L, right caudate, right angular, as well as left calcarine cortex during MwoA interictal periods.

The putamen and caudate are important part of the basal ganglia. Previous studies have found changes in basal ganglia volume, functional connectivity, and abnormal iron deposition in migraine patients [39, 40]. Our study showed that putamen and caudate CBF connection were abnormal and supported the role of basal ganglia in migraine patients.

The angular gyrus is considered to be the connecting hub of global information integration and an important part of the default mode network (DMN) [41]. Studies have shown that the angular gyrus functional connection is abnormal in migraine with visual aura [42]. In visual snow patients, there is an enhanced resting state functional connection between the prefrontal lobe and the angular gyrus [43]. Consistently with previous studies, we also identified decreased CBF disconnections between the ORBmid.R and the angular gyrus. These studies showed that the angular gyrus significantly contributes to visual information processing and the sensory cortical network.

Much of the primary visual cortex (BA 17) is hidden from view within the banks of the calcarine sulcus [44]. Significantly increased functional connectivity between the right thalamus and the left calcarine cortex was reported in the study of Wei et al., which also showed a positive correlation between the neural activation of the left calcarine cortex and the visual analogue scale scores [45]. The FC between the cerebellum and the left calcarine cortex also increases in migraine patients [46]. In line with previous findings, we also identified CBF disconnections between ORBmid.R and the left calcarine cortex. Photophobia is a common accompanying symptom in migraine patients, while visual abnormality is the most common aura symptom in migraine patients with aura [10]. The abnormal CBF connectivity of the calcarine cortex and angular gyrus can help us better understand the pathophysiological basis of photophobia in migraine patients. Accordingly, the disconnection between ORBmid.R and these areas may be related to functional defects in optical signal processing in MwoA patients. The CBF of ORBmid.R was positively correlated with the VLSQ-8 score, which may indicate interactions between optical signal processing and the perception regulatory network.

The vermis is located within the spinocerebellum and receives somatic sensory input from the head and proximal body parts via ascending spinal pathways. Cerebellar vermis atrophy in hemiplegic migraine has been previously detected by MRI [47]. Vermis atrophy has also been found in patients with familial hemiplegia migraine (FHM) [48]. Functional MRI also revealed an increase in the apparent diffusion coefficient median values. *N*-acetyl aspartate (NAA) and glutamate (Glu), was were significantly reduced while myo-inositol (mI) was significantly elevated in the vermis in patients with FHM [49]. In the present study, we identified decreased CBF in the vermis for the first time in paroxysmal migraine. However, the function of the vermis remains unclear, which necessitates further research.

This study has certain limitations. Firstly, only interictal MwoA patients were enrolled, thus we cannot speculate on whether there is a difference between MwoA and MWA patients. Secondly, we only performed a single scan for each patient, thereby the dynamic perfusion changes during different phases of a migraine attack and post-attack were not gauged. Thus, we might repeatedly scan migraine patients at several time points in future studies. Finally, only the regions with statistical differences in CBF between groups were selected as the ROI for CBF connectivity analysis, which may lead to potential loss of data. Therefore, follow-up research will consider the whole brain CBF connection analysis method.

## Conclusions

In summary, this study used ASL-MRI to detect changes in the CBF of multiple cortical regions, which may be one of the bases for pathophysiological changes in MwoA. Abnormal CBF connectivity between ORBmid.R and the regions of right putamen, SFG.L, right caudate, right angular, as well as left calcarine cortex was revealed for the first time in MwoA. These areas involve information perception, information integration, and emotional, pain, and visual processing. These results may provide important clues for elucidating the pathophysiology of migraine. As a whole, changes in CBF and CBF connectivity emphasize the necessity of studying the underlying neuropathology of MwoA patients from the perspective of resting CBF and CBF connections.

## Data Availability

All data and materials generated in this study are available upon request.

## Abbreviations

CBF: Cerebral blood flow
MwoA: Migraine without aura
HC: Healthy control
ORBmid.R: Right middle frontal orbital gyrus
VLSQ-8: Visual Light Sensitivity Questionnaire-8
ASL: Arterial spin labeling
PCASL: Pseudo-continuous ASL
SAS: The Hamilton Anxiety Scale
SDS: The Hamilton Depression Rating Scale
MOCA: The Montreal Cognitive Assessment
HIT-6: The Headache Impact Test-6
MIDAS: The Migraine Disability Assessment Questionnaire
MFG.R: The right middle frontal gyrus
SFG.L: The left superior frontal gyrus
OFC: Orbital frontal cortex
BOLD: Blood oxygen level dependent
